# Is the Combination of ADOS and ADI-R Necessary to Classify ASD? Rethinking the “Gold Standard” in Diagnosing ASD

**DOI:** 10.3389/fpsyt.2021.727308

**Published:** 2021-08-24

**Authors:** Inge Kamp-Becker, Johannes Tauscher, Nicole Wolff, Charlotte Küpper, Luise Poustka, Stefan Roepke, Veit Roessner, Dominik Heider, Sanna Stroth

**Affiliations:** ^1^Department of Child and Adolescent Psychiatry, Psychosomatics and Psychotherapy, Philipps University, Marburg, Germany; ^2^Department of Mathematics and Computer Science, Philipps University Marburg, Marburg, Germany; ^3^Department of Child and Adolescent Psychiatry and Psychotherapy, Faculty of Medicine of the Technische Universität Dresden, Dresden, Germany; ^4^Department of Psychiatry, Charité – Universitätsmedizin Berlin, Berlin, Germany; ^5^Department of Child and Adolescent Psychiatry and Psychotherapy, University Medical Center Göttingen, Göttingen, Germany

**Keywords:** machine learning, random forest, autism spectrum disorder, clinical characteristics, differential diagnosis behavioral aspects, ADOS, ADI-R, Goldstandard

## Abstract

Diagnosing autism spectrum disorder (ASD) requires extensive clinical expertise and training as well as a focus on differential diagnoses. The diagnostic process is particularly complex given symptom overlap with other mental disorders and high rates of co-occurring physical and mental health concerns. The aim of this study was to conduct a data-driven selection of the most relevant diagnostic information collected from a behavior observation and an anamnestic interview in two clinical samples of children/younger adolescents and adolescents/adults with suspected ASD. *Via* random forests, the present study discovered patterns of symptoms in the diagnostic data of 2310 participants (46% ASD, 54% non-ASD, age range 4–72 years) using data from the combined Autism Diagnostic Observation Schedule (ADOS) and Autism Diagnostic Interview—Revised (ADI-R) and ADOS data alone. Classifiers built on reduced subsets of diagnostic features yield satisfactory sensitivity and specificity values. For adolescents/adults specificity values were lower compared to those for children/younger adolescents. The models including ADOS and ADI-R data were mainly built on ADOS items and in the adolescent/adult sample the classifier including only ADOS items performed even better than the classifier including information from both instruments. Results suggest that reduced subsets of ADOS and ADI-R items may suffice to effectively differentiate ASD from other mental disorders. The imbalance of ADOS and ADI-R items included in the models leads to the assumption that, particularly in adolescents and adults, the ADI-R may play a lesser role than current behavior observations.

## Introduction

Autism spectrum disorder (ASD) is a neurodevelopmental disorder whose symptoms emerge in early development, are present in multiple contexts and persist over the lifespan. Over time, ASD has shifted from a “childhood condition” with associated challenges in language and intellectual functioning, to a wider concept of ASD including individuals with only mild symptoms or who do not show symptoms until later in life (1). Amongst other reasons for increasing prevalence rates in all age groups (2), this adjustment in the ASD concept leads to increasing numbers of individuals undergoing ASD assessment with major implications for clinical services. Current approaches mainly extend diagnostic methods designed for use in childhood to adulthood, leaving the evaluation of adult diagnostic methods “an urgent research priority” [(3), p. 11]. We thus investigated diagnostic data from adolescents and adults in comparison to data from children and younger adolescents to extend current knowledge on adults' characteristic ASD symptoms.

The current diagnostic gold standard includes two essential components: a direct observation of behavior by an experienced clinician (Autism Diagnostic Observation Schedule, ADOS) (4, 5) and an anamnestic interview with caregivers (Autism Diagnostic Interview, Revised, ADI-R) (6). Both instruments are assumed to contribute additively to the clinical judgment and to lead to a consistent and rigorous application of diagnostic criteria (7, 8). The ADOS is conducted through a one-to-one interaction and provides direct information on current ASD symptoms. It is complemented by the ADI-R, which provides information on early development, focusing mainly on the time period between 4 and 5 years of age. Due to the lengthy nature and required in-depth training for both instruments, the usage of this gold standard is confined to specialty clinics that usually struggle with limited personnel capacities and long waiting lists for diagnostic appointments.

Despite a wealth of studies investigating ASD symptoms in toddlers and children, knowledge on behavioral ASD characteristics, as assessed by ADOS and ADI-R, that may be specific to adulthood and that differentiate ASD from other mental disorders is still sparse. Results of previous studies show difficulties of the ADOS and ADI-R to discriminate between diagnostic groups with overlapping symptoms such as schizophrenia (9, 10) or personality disorders (11, 12). Although ASD is considered a lifelong condition, developmental changes further complicate recognition of symptoms in adults (13). Symptoms and impairments vary much more strongly in adolescence and adulthood than in childhood and the diagnosis of ASD in adulthood can rely much less on “prototypes” (14).

In addition to observation of current behavior, the diagnosis of ASD relies on knowledge of developmental history, thus the clinician needs access to valid information *via* caregivers, early medical or school records, which may be increasingly difficult to retrieve with the increasing age of the individual with suspected ASD (15). The ADI-R may furthermore be subject to retrospective recall biases or may be affected by inaccurate caregiver memory, particularly if the caregiver was not concerned about their child's behavior in earlier childhood (16). This is reflected by low agreement between diagnoses based on ADI-R and those based on ADOS, particularly for older and atypical cases (7, 17–21).

These considerations hold important implications for the assessment of ASD in later adolescence and adulthood, as instruments based on what is known about childhood ASD may not be as sensitive to impairments relevant to diagnosis in older individuals. It is thus essential to further understand what the core diagnostic features are and how they are best assessed in adulthood. One recent attempt to identify patterns of core information for a diagnostic decision makes use of machine-learning methods investigating the ADOS (22–24), the ADI-R (25, 26) or other sources of information, such as screening instruments or home videos (27, 28). The combination of ADOS and ADI-R data has not yet been studied. The aim of the present study was the characterization of those items from the combined ADOS and ADI-R that perform best in classifying ASD vs. non-ASD in subsamples of children and younger adolescents (ADOS module 3 and ADI-R data) and adolescents and adults (ADOS module 4 and ADI-R data). Furthermore, we aimed to investigate whether classifiers including the core diagnostic features yield better discriminative power than classifiers including only information from the ADOS, and whether reduced subsets of diagnostic features may be sufficient to validly classify ASD and non-ASD cases.

## Materials and Methods

### Participants

The presented project is part of the ASD-Net, a large consortium for the research on ASD (29). To assemble a representative sample of individuals who seek an investigation of ASD, the presence of a clinical suspicion of ASD was the general inclusion criterion. The sample incorporates *N* = 2,307 cases of children, adolescents and adults. ADOS data were available for *N* = 2,288 individuals and ADI-R data were available for *N* = 1,258 individuals. Analogous to clinical practice, the data set was divided into two subsamples based on the patients' expressive language level and chronological age, suiting the chosen ADOS module: Module 3 for children and younger adolescents (average age: 10.2, average IQ: 99.15); Module 4 for adolescents and adults (average age: 26.8, average IQ: 102.13). The two data sets were investigated separately and are henceforth labeled children/younger adolescents (ADOS Module 3 with associated ADI-R data) and adolescents/adults (ADOS Module 4 with associated ADI-R data). All subjects were classified as ASD or non-ASD cases based on best-estimate clinical (BEC) diagnosis according to ICD-10, comprising a comprehensive clinical investigation with physical examination, medical history-taking, assessment of intellectual ability, ADOS, ADI-R and differential diagnostic examination.

An ASD diagnosis was determined in 46% (*N* = 1,073) of the sample, of which 40% (*N* = 433) had comorbid disorders. Despite an initial suspicion of ASD, *N* = 1,234 individuals received either a diagnosis of a mental disorder other than ASD (*N* = 898) or no mental disorder, but developmental delays (*N* = 336). This non-ASD group represents a well-balanced clinical group, comprising different mental disorders as well as individuals without mental disorders but with some symptoms of ASD (“autistic traits”), but no complete fulfillment of ASD criteria. Participants' characteristics are presented in detail in Table 1. Further details on the psychopathology of both subsamples are provided in the Supplementary Tables 1, 2.

**Table 1:** Sample characteristic for the two subsamples of children/younger adolescents (ADOS module 3 and associated ADI-R data) and adolescents/adults (ADOS module 4 and associated ADI-R data).

	**ASD**		**Non-ASD**		***t*** **-test**			
	***N***	***M* (SD)**	***N***	***M* (SD)**	***t***	**df**	***p***	**ES**
**Children/younger adolescents**								
Age	558	10.43 (2.87)	805	9.92 (2.64)	−3.39	1,361	0.001	0.18
IQ	438	100.8 (20.1)	519	97.5 (27.6)	−2.11	955	0.035	0.13
IQ-Level	474	3.09 (.86)	737	3.09 (.87)	−0.020	1,209	0.984	0.01
SA	547	9.76 (4.06)	802	3.14 (3.49)	−32.01	1,347	0.000	1.78
RRB	547	1.44 (1.36)	802	0.31 (0.61)	−20.75	1,347	0.000	1.15
SA+RRB	547	11.20 (4.59)	802	3.45 (3.67)	−34.33	1,347	0.000	1.90
**Adolescents/adults**								
Age	515	26.27 (11.2)	429	27.41 (12.2)	1.49	942	0.13	0.09
IQ	227	104.78 (22.6)	150	99.49 (22.4)	−2.23	375	0.027	0.24
IQ-Level	472	2.87 (.78)	389	2.92 (.71)	0.926	859	0.355	0.06
SA	513	9.83 (4.23)	426	3.93 (3.69)	−22.79	937	0.000	1.50
RRB	513	1.53 (1.33)	426	0.58 (.85)	−12.78	937	0.000	0.84
SA + RRB	513	11.36 (4.94)	426	4.51 (4.13)	−22.78	397	0.000	1.49

*IQ, intelligence quotient; IQ-Level, IQ-level according to ICD-10 categories; SA, social affect domain (ADOS); RRB, restricted and repetitive behaviors domain (ADOS); SA + RRB, total scores from the ADOS algorithms*.

Participants' data were collected retrospectively from the medical records of the respective clinic (retrospective chart review) and analyzed anonymously. The procedure was approved by the local ethics committee (Az. 92/20) and due to the retrospective nature of data collection and analysis based on anonymized data, the need for informed consent was waived by the ethics committee. All methods were performed in accordance with the relevant institutional and international research guidelines and regulations.

### Measures

The ADOS is an internationally used diagnostic instrument that consists of four modules to be administered on the basis of the individual's level of expressive language and chronological age and the appropriateness of assessment materials and a module for toddlers (5). Each module provides different tasks, including playful elements and activities as well as verbal tasks intended to provide the examiner with information about social, communicative, play and stereotyped behavior. All modules provide a scoring algorithm comprising subsets of 11 items for modules 3 and 4 that have been identified as diagnostically most relevant. The ADI-R is a structured anamnestic clinical caregiver interview that mostly focuses on ASD-related symptoms at the age of 4.0–5.0 years (6). The diagnostic algorithm is organized into three behavioral domains: qualitative abnormalities in reciprocal social interaction; qualitative abnormalities in communication; and restricted and repetitive behavior (RRB). The interview contains 93 items of which 37 are used in the classification algorithm.

### Data Preparation

ADOS codes are basically indicative of symptom severity by coding increasing severity *via* codes of 0, 1, 2, and 3. Certain ADOS codes additionally contain information about peculiar or abnormal behavior *via* codes of 7 or 8. Following the ADOS manual instructions, we remapped 7 and 8 codes to 0 and codes of 3 were recoded to 2. All ADOS items were included in the machine-learning procedure. For ADI-R, data preparation and recoding were carried out similarly. Only the 37 algorithm items were included in the analysis without domain D (Abnormality of Development Evident at or Before 36 months), as these items do not address symptomatology of ASD. A list of all included items and their abbreviations can be found in Supplementary Table 3.

### Machine Learning

Previous classification studies have applied a multitude of machine-learning techniques. We chose a random forest (RF) which is robust against noise, outliers and overlapping target classes (which may well be the case in our BEC set of data) (30) and can very well be used to identify the most important features among all features available in the data set (31). The random forest consists of a collection of tree-structured classifiers constructing a multitude of decision trees at training time. Each decision tree yields a class prediction considering a random subset of features, and the consensus vote of all the trees (“the forest”) forms the final classification (30). To address the above-mentioned research questions, we built random forests with (a) the combination of ADOS and ADI-R data and (b) ADOS data alone. Modeling was performed for the two subsamples of children/young adolescents and adolescents/adults separately.

To validate each model's accuracy, a portion of 25% of the data set was left out during algorithm training and served as a validation data set. During the creation of the models, a 20-fold cross-validation was applied using 95% of the data for training and 5% for testing. Missing values were treated as valid values, i.e., all cases were used for the computations of the training and the test models. The level of significance was set at *p* ≤ 0.05. For each set of data an optimal model was chosen according to the area under the ROC curve (AUC). Utilizing the Youden Index, which incorporates sensitivity and specificity, the optimal threshold (where the AUC is at its maximum) was identified. The Youden Index is a way of summarizing the performance of a diagnostic test evaluating its discriminative power (32). The index was calculated for each threshold of the ROC curve, and the point where it achieved a maximum is referred to as the “optimal” threshold. At this particular threshold, the models' accuracy (ACC), sensitivity and specificity were evaluated and are presented as indices of model quality.

Our approach comprised four consecutive steps. First, to create a hierarchy of importance for the features, the RF permutation-based feature importance scores were used, based on 20 RFs consisting of 400 decision trees each. A 20-fold cross-validation was run on the training data. By saving every run's importance hierarchy, each features rank was identified. In a second step, a training of reduced feature models for 1 to *n* sets of features ({1},{1,2},{1,2,3}…{1,2,…n}) was undertaken, entering features to the model according to their place in the feature importance ranking—where *n* is the number of all features in the data set. To examine the optimal number of features, the resulting *n* models were compared by using both AUC and balanced accuracy (ACC) given the Youden Index determined in the prior cross-validation process during training. This represents the one point on the ROC curve for which the distance to the chance line is maximal and thus leads to the best classification result that is least likely to happen by chance. The point also represents the class boundary and is thus integrated in the subsequently created models as the threshold for decision-making. After computing the AUC and balanced ACC for the *n* models, yet another hierarchy ordering these results was established, based on the idea of information criteria, such as Akaike (AIC) and Bayesian information criterion (BIC) to determine the best performing model: Each model's classification performance (AUC) and its number of features were scaled to the unit interval, then weighted and summed, resulting in an individual score for each model. In order to identify simple models with still sufficient classification performance, we emphasized less complex models in a 2:1 ratio (i.e., w1^*^AUC + w2^*^complexity where w1 = 0.35 and w2 = 0.65). Reduced feature models were then ranked according to their weighed scores and the best performing model (simple but with good performance) could be identified as the “optimal model.”

In a third step, we tested the reduced-feature models on the hitherto unseen validation data set with regards to their classification performance. The fourth step was the comparison of the predictive performance of the reduced-feature models. We used the McNemar test, a non-parametric statistical test for paired comparisons, which can be applied to compare the performance of two machine-learning classifiers (33). All models including *n* + 1 features were evaluated regarding differences in classification error rates compared to the full-feature model. We then identified (a) the “optimal model,” comprising the optimal number of features against the “full-feature model” for both databases (combined ADOS and ADI-R data, and ADOS data alone), respectively. This was complemented by (b) the search of a “minimal-feature model,” which contained as many features as needed to exceed the *p* = 0.05 threshold of significant differences in classification error rates compared to the “full-feature-model.”

## Results

For an overview of the model's performances and the comparisons of the respective features refer to Table 2. Table 3 gives an overview of the features selected by the classifier.

**Table 2:** Performance of the machine-learning models on the test set and the previously unseen validation data set for the two subsamples of children/younger adolescents (ADOS module 3 and associated ADI-R data) and adolescents/adults (ADOS module 4 and associated ADI-R data).

**Sample**	**No. of features**	**AUC test**	**ACC test**	**Sens. test**	**Spec. test**	**Youden's J**	**AUC blind**	**ACC blind**	**Sens. blind**	**Spec. blind**	**McNe**
Children/younger	ADOS + ADI-R combination										
adolescents	All 65 features	0.91	0.88	0.89	0.82	0.54	0.94	0.87	0.92	0.81	1
	11 features (optimal model) (7 ADOS, 4 ADI-R)	0.90	0.87	0.88	0.79	0.48	0.93	0.85	0.93	0.78	0.81
	7 features (minimal model) (6 ADOS, 1 ADI-R)	0.86	0.84	0.86	0.80	0.51	0.80	0.82	0.85	0.79	0.11
	ADOS alone										
	All 28 features	0.93	0.89	0.92	0.86	0.41	0.92	0.85	0.90	0.80	1
	7 ADOS features (optimal model)	0.92	0.88	0.89	0.88	0.42	0.90	0.82	0.82	0.83	0.01
	9 ADOS features (minimal model)	0.92	0.88	0.89	0.88	0.41	0.90	0.82	0.88	0.81	0.15
Adolescents/adults	ADOS + ADI-R combination										
	All 68 features	0.87	0.88	0.83	0.90	0.62	0.83	0.74	0.83	0.66	1
	8 features (optimal model) (6 ADOS, 2 ADI-R)	0.83	0.83	0.84	0.81	0.62	0.77	0.70	0.79	0.62	0.20
	7 features (minimal model) (5 ADOS, 2 ADI-R)	0.84	0.86	0.85	0.83	0.61	0.79	0.68	0.83	0.53	0.08
	ADOS alone										
	All 31 features	0.83	0.84	0.86	0.82	0.52	0.90	0.82	0.90	0.74	1
	5 ADOS features (optimal model)	0.85	0.83	0.84	0.82	0.52	0.85	0.75	0.87	0.63	0.01
	8 ADOS features (minimal model)	0.82	0.82	0.90	0.73	0.46	0.78	0.73	0.90	0.58	0.34

*No. of features, number of features entered in the model; AUC, area under the curve; ACC, accuracy; Sens., sensitivity; Spec., specificity; J, Youden's Index; McNe, McNemar level of significance—each model tested against the whole set of available features in the combination of ADOS, and ADI and ADOS, respectively. The “optimal model” represents the model with optimal relation of model performance and complexity. The “minimal model” represents the model with the least number of variables that does not significantly differ from the full-feature model as asserted by the McNemar test*.

**Table 3:** The optimal number of features for the combined data (ADOS + *ADI-R*) for children/younger adolescents (ADOS module 3 and associated ADI-R data) and adolescents/adults (ADOS module 4 and associated ADI-R data) (upper row left and right).

**Chirdren/younger adolescents** **ADOS + *ADI-R***	**Adolescents/adults** **ADOS + *ADI-R***
1. Quality of social overtures (ADOS) 2. Speech abnormalities associated with autism (ADOS) 3. Facial expressions directed to examiner (ADOS) 4. Amount of reciprocal social communication (ADOS) 5. Stereotyped/idiosyncratic use of words or phrases (ADOS) 6. Conversation (ADOS) 7. *Reciprocal conversation (*ADI-R) 8. Insight into typical social situations and relationships (ADOS) 9. *Imitative social play* (ADI-R) 10. *Interest in children* (ADI-R) 11. *Showing/directing attention* (ADI-R)	1. Facial expressions directed to examiner (ADOS) 2. Unusual eye contact (ADOS) 3. Quality of social responses (ADOS) 4. Speech abnormalities associated with autism (ADOS) 5. Descriptive, conventional, instrumental or informational gestures (ADOS) 6. *Showing/directing attention* (ADI-R) 7. *Pointing to express interest* (ADI-R) 8. Quality of social overtures (ADOS)
**Chirdren/younger adolescents** **ADOS**	**Adolescents/adults** **ADOS**
1. Amount of reciprocal social communication 2. Stereotyped/idiosyncratic use of words or phrases 3. Conversation 4. Quality of social overtures 5. Facial expressions directed to examiner 6. Insight into typical social situations and relationships 7. Descriptive, conventional, instrumental or informational gestures	1. Quality of social responses 2. Comments on other's emotions/empathy 3. Quality of social overtures 4. Amount of reciprocal social communication 5. Unusual eye contact

*The optimal number of features for the behavior observation (ADOS) for Children/Younger Adolescents (ADOS Module 3 and associated ADI-R data) and Adolescents/Adults (ADOS Module 4 and associated ADI-R data) (lower row left and right)*.

### ADOS in Combination With ADI-R Data in Children/Younger Adolescents

**Figure 1:**
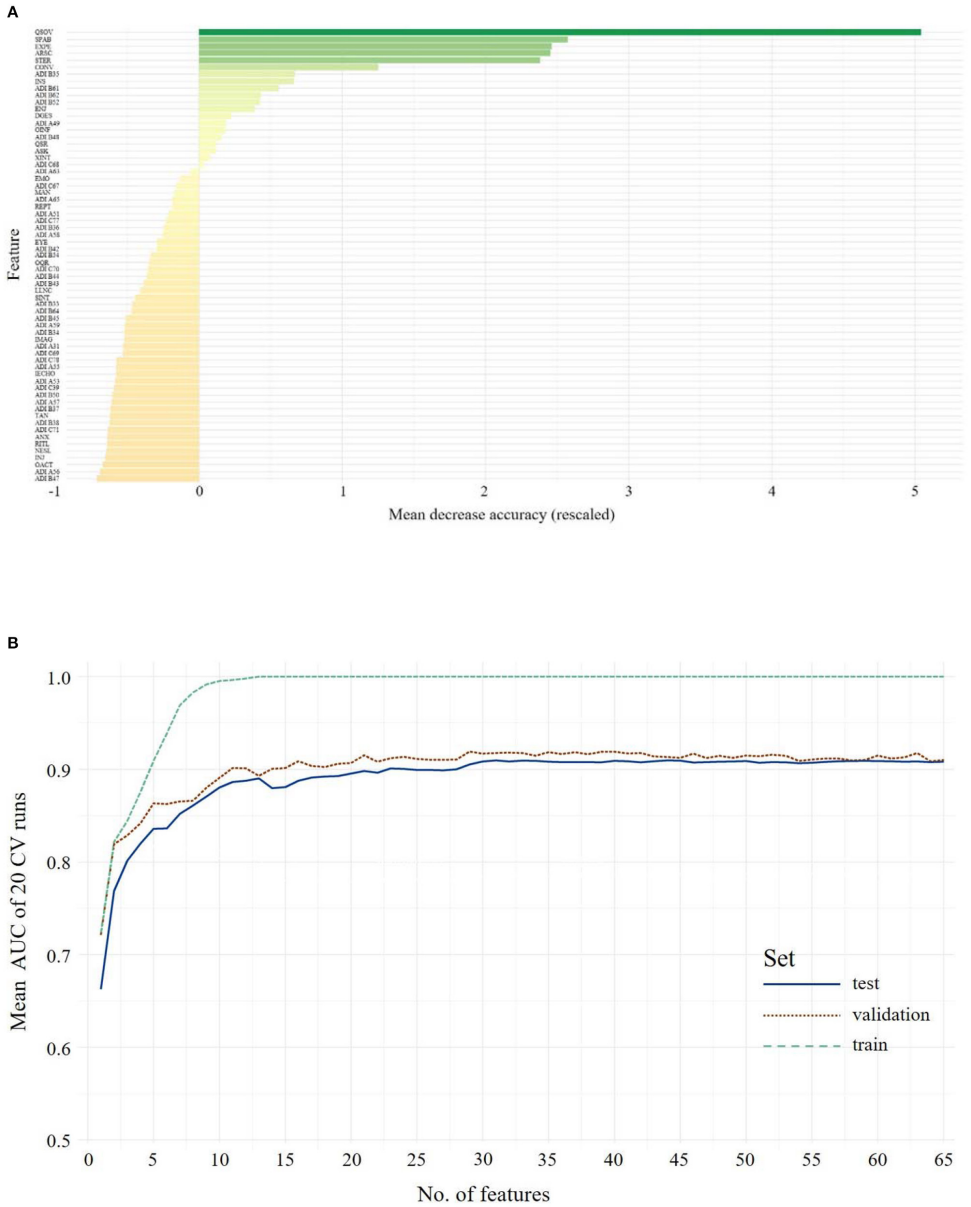
The upper panel shows the overall ranking of feature importance for all features from **ADOS and ADI-R** data combined for **Children/Younger Adolescents (A)**. The figure depicts the ADOS and ADI items on the y-axis and on the x-axis its corresponding importance score, measured in mean decrease in accuracy. The lower panel **(B)** shows the mean AUC plotted against the number of model features from **ADOS and ADI-R** combined during model building (training, testing and validation of the classifiers) for **Children/Younger Adolescents**. A list of all included features and their abbreviations can be found in Supplementary Table 3.

By utilizing the importance hierarchy shown in Figure 1A (larger versions of the figures can be found in Supplementary Figures S1–S4), RFs for 1 to *n* features were calculated and tested. The model output from the test set including all 65 features shows an ACC of 0.88, with 0.89 sensitivity and 0.82 specificity. For independent validation of the classifier, its performance on the validation data set was computed showing a stable performance, with an ACC of 0.87 and 0.92 sensitivity and 0.81 specificity. The feature selection vs. performance curve in Figure 1B shows that only few features contribute strongly to the class prediction, whereas others seem to have very little predictive value. The model including 11 features showed optimal performance in the validation set: The ACC is 0.85, with 0.93 sensitivity and 0.78 specificity. This model includes seven features from the ADOS and four from the ADI-R. McNemar's test for differences in classification error rates showed no advantage of the full-feature model (65 features) over the 11-feature model (χ^2^ = 0.06, *p* = 0.81). The optimal model included the following features: Quality of Social Overtures (ADOS), Speech Abnormalities Associated With Autism (ADOS), Facial Expressions Directed to Examiner (ADOS), Amount of Reciprocal Social Communication (ADOS), Stereotyped/ Idiosyncratic Use of Words or Phrases (ADOS), Conversation (ADOS), Reciprocal Conversation (ADI-R), Insight Into Typical Social Situations and Relationships (ADOS), Imitative Social Play (ADI-R), Interest in Children (ADI-R), Showing/Directing Attention (ADI-R). This already reduces the feature set, however as Figures 1A,B^1^ suggest, there might be even more potential for a reduction in the coding systems. We thus searched for the minimal model, whose prediction error is statistically equal to the full-feature model. McNemar's test showed that a seven-feature model was the one with the least number of features that did not differ from the full-feature model in the validation set (χ^2^ = 2.50, *p* = 0.11; ACC = 0.82, sensitivity = 0.85, specificity = 0.79).

### ADOS in Combination With ADI-R Data in Adolescents/Adults

**Figure 2:**
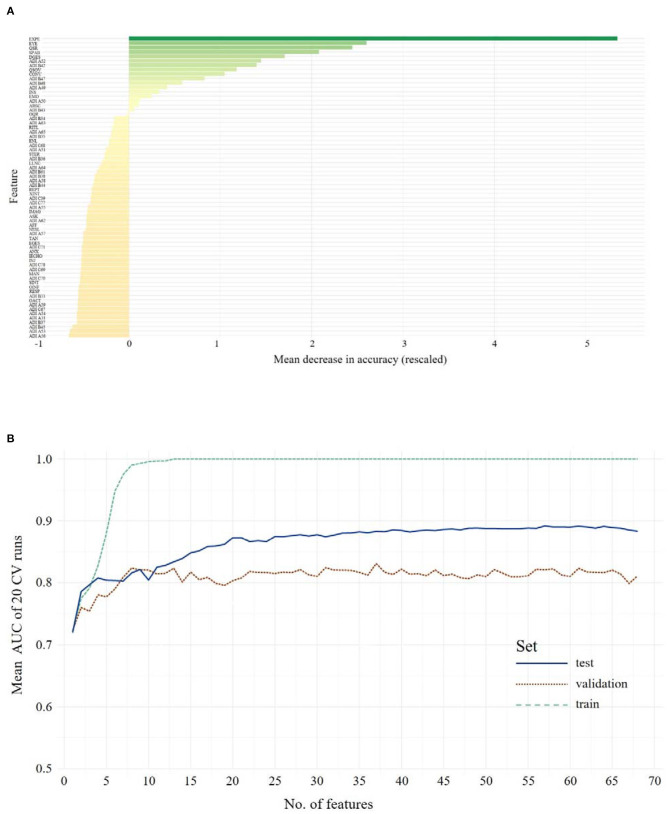
The upper panel shows the overall ranking of feature importance for all features from **ADOS and ADI-R** data combined for **Adolescents/Adults (A)**. The figure depicts the ADOS and ADI items on the y-axis and on the x-axis its corresponding importance score, measured in mean decrease in accuracy (see text footnote 1). The lower panel **(B)** shows the mean AUC plotted against the number of model features from **ADOS and ADI-R** combined during model building (training, testing and validation of the classifiers) for **Adolescents/Adults**. A list of all included features and their abbreviations can be found in Supplementary Table 3.

A feature selection for the combined ADOS and ADI-R data was performed, resulting in an overall ranking of feature importance shown in Figure 2A^1^. Again, RFs for 1 to *n* features were calculated and evaluated in the validation data set. The full-feature model, including the combination of 31 ADOS items and 37 ADI-R algorithm items showed an ACC of 0.88 and 0.83 sensitivity and 0.90 specificity in the training set. Validating the full-feature model in an independent validation data set yielded an ACC of 0.74, with 0.83 sensitivity and 0.66 specificity. The mean AUC increases when more features are used for training, but soon reaches a classification performance that does not further improve with more features (see Figure 2B). We thus examined performances of reduced feature models with eight features (identified as the optimal number of features by the Youden Index) in the validation set, yielding an ACC of 0.70, with 0.79 sensitivity and a specificity of 0.62. The following features were identified: Facial Expressions Directed to Examiner (ADOS), Unusual Eye Contact (ADOS), Quality of Social Responses (ADOS), Speech Abnormalities Associated With Autism (ADOS), Descriptive, Conventional, Instrumental or Informational Gestures (ADOS), Showing/Directing Attention (ADI-R), Pointing to Express Interest (ADI-R), Quality of Social Overtures (ADOS). Statistical comparison *via* McNemar's tests showed no advantage of the full-feature model over the eight-feature model (χ^2^ = 1.66, *p* = 0.20). The minimal model contained seven features (ACC = 0.68, sensitivity = 0.83, specificity = 0.53) and did not differ from the full-feature model regarding classification error rates (χ^2^ = 3.16, *p* = 0.08).

### ADOS Data Children/Younger Adolescents

**Figure 3:**
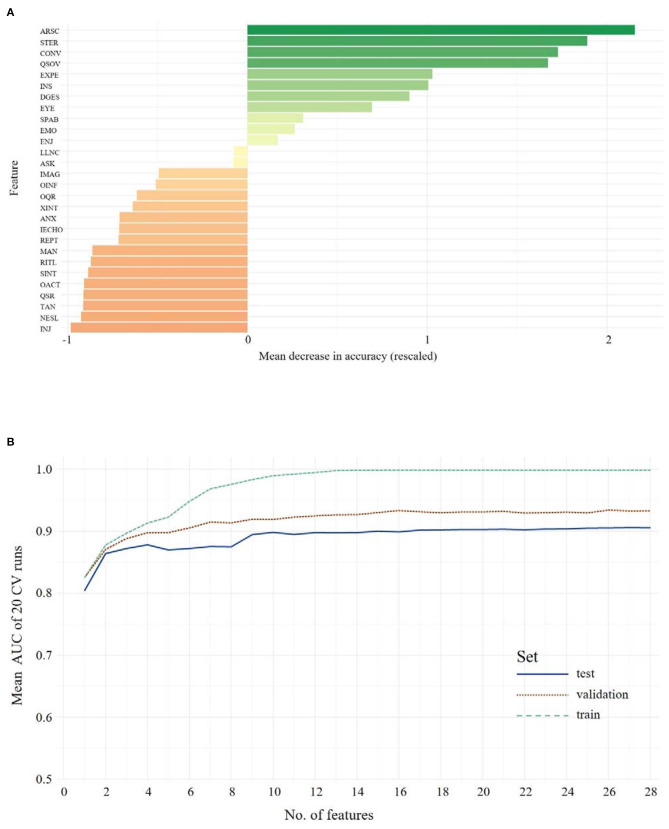
The upper panel shows the overall ranking of feature importance for all features from **ADOS** data for **Children/Younger Adolescents (A)**. The figure depicts the ADOS and ADI items on the y-axis and on the x-axis its corresponding importance score, measured in mean decrease in accuracy (see text footnote 1). The lower panel **(B)** shows the mean AUC plotted against the number of model features from **ADOS** during model building (training, testing and validation of the classifiers) for **Children/Younger Adolescents**. A list of all included features and their abbreviations can be found in Supplementary Table 3.

The same RF approach was carried out with ADOS data of children/younger adolescents. First, a feature importance hierarchy was established (see Figure 3A)^1^. In order to identify the optimal number of features, RFs including 1 to *n* features were trained and the models were evaluated in the validation data set. As shown in Figure 3B, the mean AUC increases when more features are used for training but soon reaches a plateau. The model, including all 28 ADOS items, showed an ACC of 0.89, with 0.92 sensitivity and 0.86 specificity. Evaluated on the validation data set, performance of the classifier dropped only slightly to an ACC = 0.85, with 0.90 sensitivity and 0.80 specificity. The optimal number of features (Youden Index = 0.405) was seven features from the ADOS. With only seven features, the classifier achieved an ACC of 0.88, 0.89 sensitivity and 0.88 specificity in the test set and an ACC of 0.82, 0.82 sensitivity and 0.83 specificity in the validation set. These seven features were identified: Amount of Reciprocal Social Communication, Stereotyped/Idiosyncratic Use of Words or Phrases, Conversation, Quality of Social Overtures, Facial Expressions Directed to Examiner, Insight Into Typical Social Situations and Relationships, Descriptive, Conventional, Instrumental or Informational Gestures. Statistical comparison of the models *via* McNemar's test of differences between classification error rates still showed the advantage of the full-feature model over the seven-feature model (χ^2^ = 7.23, *p* = 0.007). Only when nine features were used for the model did the statistical comparison not yield a significant advantage of the full full-feature model (χ^2^ <2.1, *p* > 0.15). Thus, the nine-feature model was identified as the minimal model (ACC = 0.82, sensitivity = 0.88, specificity = 0.81).

### ADOS Data Adolescents/Adults

**Figure 4:**
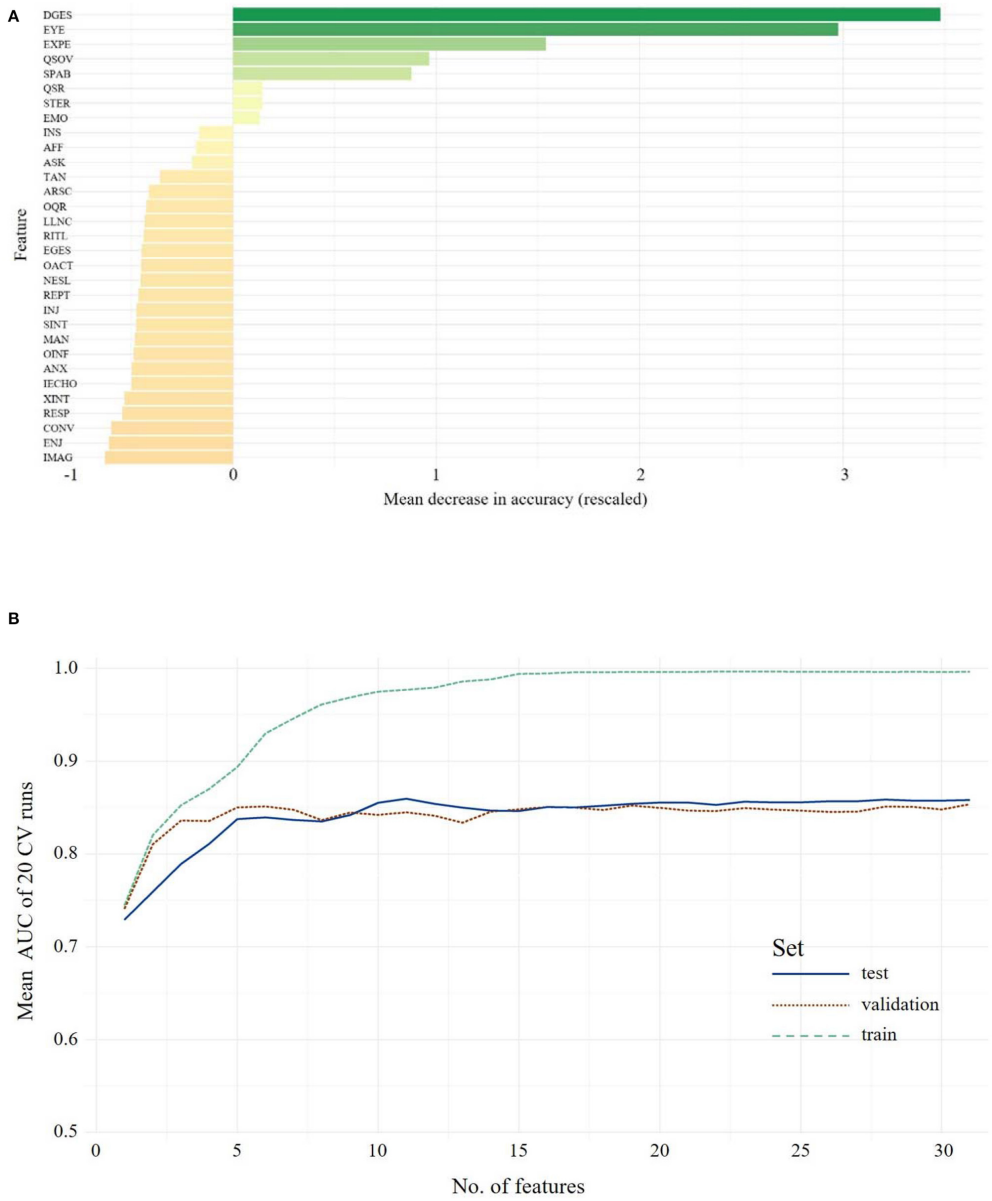
The upper panel shows the overall ranking of feature importance for all features from **ADOS** data for **Adolescents/Adults (A)**. The figure depicts the ADOS and ADI items on the y-axis and on the x-axis its corresponding importance score, measured in mean decrease in accuracy (see text footnote 1). The lower panel **(B)** shows the mean AUC plotted against the number of model features from **ADOS** during model building (training, testing and validation of the classifiers) for **Adolescents/Adults**. A list of all included features and their abbreviations can be found in Supplementary Table 3.

The hierarchy of features importance for 31 ADOS items is presented in Figure 4A^1^. In the test set, the full-feature model, including all 31 ADOS items, yielded an ACC of 0.84, 0.86 sensitivity and 0.82 specificity. In the validation set, the full-feature model performed comparably well: ACC = 0.82, 0.90 sensitivity and 0.74 specificity. Figure 4B, depicting the relation of the AUC and the number of features used for model training, shows a set point for performance of the classifier when up to eight features from the ADOS are used in model training. The optimal number of features in Module 4 (Youden index = 0.5205) is five, with an ACC of 0.83 and 0.84 sensitivity and 0.82 specificity. In the validation set, an ACC of 0.75 with 0.87 sensitivity and 0.63 specificity was observed. The optimal model included the following features: Quality of Social Responses, Comments on Other's Emotions/Empathy, Quality of Social Overtures, Amount of Reciprocal Social Communication, Unusual Eye Contact. Statistical comparison of the models *via* McNemar's test still showed the advantage of the full-feature model over the five-feature model (χ^2^ = 7.62, *p* = 0.005). Only when eight features were used for the model did the statistical comparison not yield a significant advantage of the full-feature model (χ^2^ <1.1, *p* > 0.29, ACC = 0.73, sensitivity = 0.90, specificity = 0.58).

## Discussion

Based on a well-characterized clinical population, the present work strives to localize those diagnostic items from a clinical behavior observation which most effectively differentiate between groups of children, adolescents and adults with ASD, and those with other mental disorders or developmental delays. Based on a machine-learning strategy, we were able to show that focusing attention on a few crucial behavioral aspects can lead to classification performances that are just as good as those using information from the full examination. For the combined ADOS and ADI-R data, the classifier performed optimally (pursuing highest accuracy with the least number of features) using 11 features in children/younger adolescents and eight features in adolescents/adults. For ADOS data alone, similar results were observed: Classifiers containing seven (children/younger adolescents) and five (adolescents/adults) features achieved optimal performance. However, the reduced ADOS-feature subsets representing the optimal models seemed to be still inferior to the full examination, as *post-hoc* statistical comparisons show. Only when two additional features were used for model building in children/younger adolescents and three features in adolescents/adults, did statistical comparisons not yield significant predictive advantages of the full-feature models over the reduced subsets of features. Nevertheless, our findings further corroborate the hypothesis that a reduction of complexity of the diagnostic procedure may be possible. Although the abbreviation of the ADOS itself by simply reducing the items seems not to be feasible, the current results may serve as a foundation on which training tools for clinicians could be developed. These training tools ought to support the decision of whether an individual with the suspicion of ASD needs to be referred to a specialized institution for a comprehensive ASD diagnosis, by drawing attention to the most relevant aspects that best distinguish ASD from other mental disorders and “autistic traits” in individuals without mental disorders.

Prediction performance of all selected models was lower in adolescents/adults than children/younger adolescents, reflecting the above-mentioned peculiarities of the adult sample that comprises mostly high-functional older individuals, diagnosed with ASD or other mental disorders rather late in life, who showed increased comorbidity rates in the ASD group but particularly the non-ASD (~50% in the ASD group and ~80% in the non-ASD group) which is in line with previous research (34). But besides co-occuring symptoms, overlapping symptoms (of mental or neurodevelopmental disorders) may also (negatively) influence the performance of a classifier as class boundaries are even more blurred when both groups share diagnostic signs. Thus, the composition of both groups regarding symptoms mental and neurodevelopmental disorders clearly hampers a valid and reliable classification.

### Comparison of the Combined Diagnostic Instruments (ADOS and ADI-R) vs. Behavior Observation (ADOS) Only

In children/younger adolescents, both classifiers from the combined ADOS+ADI-R and ADOS alone (including the optimal number of 11 and 7 features with only 4 and 1 ADI-R features, respectively) performed similarly well. In adolescents/adults, the classifier built upon the ADOS alone, performed even better than the classifier from ADOS+ADI-R combined (which included only two ADI-R items). These observations suggest that particularly for older adolescents and adults, information about developmental history may play a lesser role than current behavior observations. Although an ASD diagnosis requires symptoms to be present from early childhood onwards, it may be debated whether an anamnestic interview with parents of caregivers, struggling to provide details about early developmental time periods for adults, should be considered part of a gold standard. Indeed, particularly in adults, information on early symptoms are crucial and therefore a case history provided by a third party is essential. However, fine-grained anamnestic data might not be available, sufficiently detailed or might be inaccurate due to the long time lag and may thus be vulnerable to several biases (recall- or confirmation-bias, halo-, contrast- or expectancy-effects, social desirability, etc.) reducing the validity of retrospective statements (35–38). According to the DSM-5, the examiner has to ensure that no evidence for appropriate social or communicative abilities during childhood exist as a report of normal and reciprocal friendships or communicative non-verbal behavior in childhood would rule out the diagnosis of ASD. Where informants, who were present in childhood, are not available, or recall seems biased, clinicians need to seek other informants, such as older siblings, relatives or friends who knew the patient well as a child, school reports, or—wherever possible—observations of informants who have known the patient in adulthood. Although the present results suggest that, particularly for adolescents/adults, the ADI-R may be of minor importance compared to the ADOS, other studies identified the ADI-R as an appropriate instrument to accurately predict symptom severity for certain individuals (39).

### Differences of Core Diagnostic Features Between Children/Younger Adolescents and Adolescents/Adults

Since certain behaviors or symptoms follow a particular developmental course, the coupling of age and certain “core” features may increase the capacity of clinicians to recognize characteristic autistic behavior. In the present study we find a few overlapping features between the age groups: ADOS: “Facial Expressions Directed to Examiner” (EXPE), “Speech abnormalities associated with autism” (SPAB), “Quality of social overtures” (QSOV); ADI-R: “Showing and Directing Attention.” Other aspects differ between the age groups: While for adolescents/adults “Unusual Eye Contact” (EYE) and “Comments on Other's Emotions/Empathy” (EMO) are important items, for children/younger adolescents these features are less relevant, but “Stereotyped/ Idiosyncratic use of words or phrases” (STER) and “Conversation (CONV) are more important. An interesting result is, that developmental changes are accompanied by changes in feature combinations, but in every model non-verbal behavior—especially “Facial Expressions Directed to Others”—plays an important role and ranks amongst the six most important features. This is in accordance with increasing evidence that individuals with ASD display facial expressions less frequently and are less likely to share facial expressions with others, especially in natural contexts (40). This is also in line with prospective, longitudinal studies showing that non-verbal behavior deficits in individuals with ASD are stable over time (41) and are evident in normal-intelligence adult patients with ASD (42).

The present findings also relate to results from previous work by Bishop et al. (43), who conducted a factor analytical study showing three differentiable subdimensions of social-communication impairment in ASD: “‘Basic Social-Communication' behaviors (e.g., Facial Expressions, Unusual Eye-Contact, Gestures etc.), ‘Interaction Quality' (including more complex aspects of social-communication e.g., Conversation, Amount of Reciprocal Social Communication) and ‘Restricted and Repetitive Behaviors' (RBB).” The authors conclude that while impairments in Basic Social Communication reflect “core” impairments in ASD, they seem to be “remarkably intact” in children without ASD (but with other disorders) and thus contributed particularly well to the prediction of ASD (43).

In sum, our analyses indicate that more is not necessarily better and that a reduction of the gold-standard diagnostic procedure is possible. Different (age) groups may require a particular focus on particular aspects of the overall symptomatology leading to a particular combination of features to assess. This is in line with several other studies using different machine-learning techniques and finding slightly overlapping and different item constellations of features using either ADI-R or ADOS data (22–24, 26). Due to the fact that ADOS and ADI-R items are not independent from each other and a multitude of information adding to different aspects of behavior forms an overall picture of a patient—highly dependent on the observer—results of all machine-learning methods will be inevitably inconsistent. It has been argued that administration times for ADOS and ADI-R cannot be reduced due to the observational nature of the instruments that allow for behavior coding independent of certain tasks and thus making an abbreviation of the whole exam length impossible. However, recent research results have shown that a much briefer, unstructured social interaction, a home-video sequence or even the reliance on written extracts of children's medical and educational records may well suffice for valid coding of abnormal behavior associated with ASD (44–48).

Future research has to consider whether a reduced set of items will lead to sufficiently reliable and valid diagnostic decisions with regard to the question of whether the suspicion of ASD is reasonable and the individual really needs specialized examination. But also, a reduction of complexity would be desirable as in clinical contexts and despite training and supervision the diagnostic accuracy of ADOS and ADI-R coding is still not particularly good (49–51). Based on our results, we would recommend that the gold standard in diagnosing ASD would include, in a first step, an abbreviated but valid examination (with the reduced set of behavior observations) to decide whether a “full standard examination” (including ADOS and full ADI-R) is necessary or not. This could reduce waiting times at specialized institutions and avoid delays in diagnosis and in the delivery of therapies.

### Strengths and Limitations

A major advantage of the present study lies within the well-balanced data set from a large and well-characterized clinical sample comprising various mental disorders. The current study thus contributes to the identification of boundaries between ASD cases and those cases that exhibit ASD-like symptoms that are, however, based on different underlying conditions.

A major limitation is that the outcome criterion (BEC of ASD vs. non-ASD) was not independent of the features used for building the prediction algorithm, thus creating a certain circularity. Although this research design may be criticized, there is currently little to no alternative as there is no independent external criterion replacing BEC. For a detailed discussion see (12). We approached the circularity problem by relying on clinical best-estimate diagnoses that included multiple sources of information beyond ADOS and ADI-R and beyond a mere classification based on ADOS and ADI-R cut-off scores.

Another limitation is the wide age range of the groups (with an approximated normal distribution ranging from 4 up to 72 years) and our sample consisted of male as well as female participants. In a first step, we simply divided our data set into two subsamples according to the chosen ADOS module. Future studies should investigate differences in specific gender and age groups (i.e., children, adolescents, young, middle and late adulthood) as well as in more specific clinical comparison groups (e.g., personality disorders, anxiety disorders, other developmental disorders).

## Conclusion

It is time to rethink the “gold standard” in diagnosing ASD, as the combination of ADOS and ADI-R is a lengthy, time-consuming procedure. After a widening of the diagnostic criteria, the integration of the autism subtypes into the ASD category and the lack of objective “ASD tests” or even objective (biological or behavioral) markers, extensive experience and expertise is needed to validly diagnose ASD. Together with an increasing number of individuals demanding a diagnosis, this leads to increasingly long waiting lists at specialized institutions. Our data support the idea that in children, adolescents and adults with a suspicion of ASD the diagnostic process can be organized more efficiently. The current study identified reduced subsets of ADOS and ADI-R items that may be particularly effective in differentiating ASD from other mental disorders. Implementing these findings into training tools that instruct clinicians to focus attention on specific disorder-related aspects may facilitate the decision of whether a patient needs to be referred to a specialized institution for a comprehensive ASD diagnosis (including the complete ADOS and ADI-R) or be closely examined for general developmental delays or other mental disorders.

## Data Availability Statement

The data analyzed in this study is subject to the following licenses/restrictions: Data are not publicly available as they contain clinical information. Within the limits of cooperation projects, terms of use can be discussed. Requests to access these datasets should be directed to stroth@staff.uni-marburg.de.

## Ethics Statement

The studies involving human participants were reviewed and approved by the Ethics Committee of the University of Marburg (Az. 92/20). Due to the retrospective nature of data collection and analysis based on anonymized data, the need for informed consent was waived by the Ethics Committee. Written informed consent from the participants' legal guardian/next of kin was not required to participate in this study in accordance with the national legislation and the institutional requirements.

## Author Contributions

SSt and IK-B drafted the manuscript. NW, SSt, and CK managed the data basis. JT and SSt carried out analyses. LP, SR, VR, and IK-B designed the study. All authors critically reviewed the manuscript drafts, and approved the final manuscript.

## Conflict of Interest

LP has received payment for consulting or speaking fees from Shire, Takeda, Roche, and InfectoPharm. She receives research funding from the BMBF, DFG, and EU and royalties from Hogrefe, Kohlhammer and Schattauer. VR has received payment for consulting and writing activities from Lilly, Novartis, and Shire Pharmaceuticals, lecture honoraria from Lilly, Novartis, Shire Pharmaceuticals, and Medice Pharma, and support for research from Shire Pharmaceuticals and Novartis. He has carried out clinical trials in cooperation with the Novartis, Shire, Servier, and Otsuka companies. The remaining authors declare that the research was conducted in the absence of any commercial or financial relationships that could be construed as a potential conflict of interest.

## Publisher's Note

All claims expressed in this article are solely those of the authors and do not necessarily represent those of their affiliated organizations, or those of the publisher, the editors and the reviewers. Any product that may be evaluated in this article, or claim that may be made by its manufacturer, is not guaranteed or endorsed by the publisher.

## Footnotes

^1^The values have been rescaled for visual interpretability. With 0 being the average scaled decrease of accuracy, a variable is decreasing the model's accuracy more than average if it is greener (more positive), meaning it is more important to keep it in the model, and decreasing the model's accuracy less than average the more red (more negative) it is, meaning the variable can be omitted without loss greater than average to the model's performance.
