# Supplementary material for: Is the Combination of ADOS and ADI-R Necessary to Classify ASD? Rethinking the “Gold Standard” in Diagnosing ASD
Source: Front Psychiatry. 2021 Aug 24;12:727308. doi: 10.3389/fpsyt.2021.727308 (PMC8421762; doi:10.3389/fpsyt.2021.727308)
Supplement: Supplementary file 1 [file Data_Sheet_1.pdf]

Supplementary information

Table S1. Psychopathological characterization of Children/Younger Adolescents in the ASD and non-ASD groups. The table presents absolute numbers with percent in brackets per group.
ASD= autism spectrum disorder; ICD-10=International Statistical Classification of Diseases and Related Health Problems 10th Revision.

**Psychopathological characterization**		**ASD (n=556)**	**non-ASD (n=805)**
Cases with further (axis one or two) ICD-10 diagnoses		n=269 (48%)	n=673 (83%)
F10–F19	mental and behavioral disorders due to psychoactive substance use	1 (0.2)	0 (0.0)
F20–F29	Schizophrenia, schizotypal and delusional disorders	4 (0.7)	4 (0.5)
F30–F39	Affective disorders	0 (0.0)	3 (0.4)
F40–F48	Neurotic, stress-related and somatoform disorders	10 (1.8)	24 (3.0)
F50–F59	Behavioral syndromes associated with physiological disturbances and physical factors	0 (0.0)	3 (0.4)
F60–F69	Disorders of adult personality and behavior	1 (0.2)	5 (0.6)
F80–F89 *except* F84	Disorders of psychological development other than pervasive developmental disorders	142 (25.5)	256 (31.8)
F90–F98	Behavioral and emotional disorders with onset usually occurring in childhood and adolescence	183 (32.9)	756 (93.9)
Cases with no (axis one) ICD-10 diagnoses			n=189 (23%)

Table S2. Psychopathological characterization of Adolescents/Adults in the ASD and non-ASD groups. The table presents absolute numbers with percent in brackets per group.
ASD=autism spectrum disorder; ICD-10=International Statistical Classification of Diseases and Related Health Problems 10th Revision.

**Psychopathological characterization**		**ASD (n=515)**	**non-ASD (n=429)**
Cases with further (axis one or two) ICD-10 diagnoses		n=249 (48%)	n=346 (80%)
F10–F19	mental and behavioral disorders due to psychoactive substance use	16 (3.1)	13 (3.0)
F20–F29	Schizophrenia, schizotypal and delusional disorders	4 (0.8)	10 (2.3)
F30–F39	Affective disorders	132 (25.6)	84 (9.4)
F40–F48	Neurotic, stress-related and somatoform disorders	85 (16.5)	94 (21.7)
F50–F59	Behavioral syndromes associated with physiological disturbances and physical factors	3 (0.6)	10 (2.3)
F60–F69	Disorders of adult personality and behavior	21 (4.1)	95 (22.0)
F80–F89 *except* F84	Disorders of psychological development other than pervasive developmental disorders	31 (6.0)	44 (10.2)
F90–F98	Behavioral and emotional disorders with onset usually occurring in childhood and adolescence	64 (12.4)	100 (23.1)
Cases with no (axis one) ICD-10 diagnoses			n= 147 (34%)

Table S3: Items and items abbreviations of ADOS and ADI-R

**ADOS**	
ANX	Anxiety
ARSC	Amount of Reciprocal Social Communication
ASK	Asks for Information
CONV	Conversation
DGES	Descriptive, Conventional, Instrumental, or Informational Gestures
EMO	Empathy/Comments on Other's Emotions
ENJ	Shared Enjoyment in Interaction
EXPE	Facial Expressions Directed to Examiner
EYE	Unusual Eye Contact
IECHO	Immediate Echolalia
IMAG	Imagination/Creativity
INJ	Self-Injurious Behavior
INS	Insight
LLNC	Language Production and Linked Nonverbal Communication
MAN	Hand and Finger and Other Complex Mannerisms
NESL	Overall Level of Non-Echoed Language
OACT	Overactivity
OINF	Offers Information
OQR	Overall Quality of Rapport
QSOV	Quality of Social Overtures
QSR	Quality of Social Response
REPT	Reporting of Events
RITL	Compulsions or Rituals
SINT	Unusual Sensory Interest in Play Material/Person
SPAB	Speech Abnormalities Associated with Autism
STER	Stereotyped/Idiosyncratic Use of Words or Phrases
TAN	Tantrums, Aggression, Negative or Disruptive Behavior
XINT	Excessive Interest in or References to Unusual or Highly Specific Topics or Objects or Repetitive Behaviors
**ADI-R**	
ADI-R: A31	Use of Other's Body to Communicate
ADI-R: A49	Imaginative Play with Peers
ADI-R: A50	Direct Gaze
ADI-R: A51	Social Smiling
ADI-R: A52	Showing and Directing Attention
ADI-R: A53	Offering to Share
ADI-R: A54	Seeking to Share Enjoyment with Others
ADI-R: A55	Offering Comfort
ADI-R: A56	Quality of Social Overtures
ADI-R: A57	Range of Facial Expressions Used to Communicate
ADI-R: A58	Inappropriate Facial Expressions
ADI-R: A59	Appropriateness of Social Response
ADI-R: A62	Interest in Children
ADI-R: A63	Response to Approaches of Other Children
ADI-R: A64	Group Play with Peers
ADI-R: A65	Friendships
ADI-R: B33	Stereotyped Utterances and Delayed Echolalia
ADI-R: B34	Social Verbalization/Chat

ADI-R: B35	Reciprocal Conversation
ADI-R: B36	Inappropriate Questions of Statements
ADI-R: B37	Pronominal Reversal
ADI-R: B38	Neologism/Idiosyncratic Language
ADI-R: B42	Pointing to Express Interest
ADI-R: B43	Nodding
ADI-R: B44	Head Shaking
ADI-R: B45	Conventional/Instrumental Gestures
ADI-R: B47	Spontaneous Imitation of Actions
ADI-R: B48	Imaginative Play
ADI-R: B61	Imitative Social Play
ADI-R: C39	Verbal Rituals
ADI-R: C67	Unusual Preoccupations
ADI-R: C68	Circumscribed Interests
ADI-R: C69	Repetitive Use of Objects or Interest in Parts of Objects
ADI-R: C70	Compulsions/Rituals
ADI-R: C71	Unusual Sensory Interests
ADI-R: C77	Hand and Finger Mannerisms
ADI-R: C78	Other Complex Mannerisms or Stereotyped Body Movements
